# Association Between Automotive Assembly Plant Closures and Opioid Overdose Mortality in the United States: A Difference-in-Differences Analysis

**DOI:** 10.1001/jamainternmed.2019.5686

**Published:** 2019-12-30

**Authors:** Atheendar S. Venkataramani, Elizabeth F. Bair, Rourke L. O’Brien, Alexander C. Tsai

**Affiliations:** 1 Department of Medical Ethics and Health Policy, Perelman School of Medicine, University of Pennsylvania, Philadelphia; 2 Leonard Davis Institute of Health Economics, University of Pennsylvania, Philadelphia; 3 Department of Sociology, Yale University, New Haven, Connecticut; 4 Center for Global Health, Massachusetts General Hospital, Boston; 5 Harvard Medical School, Boston, Massachusetts

## Abstract

**Importance:**

Fading economic opportunity has been hypothesized to be an important factor associated with the US opioid overdose crisis. Automotive assembly plant closures are culturally significant events that substantially erode local economic opportunities.

**Objective:**

To estimate the extent to which automotive assembly plant closures were associated with increasing opioid overdose mortality rates among working-age adults.

**Design, Setting, and Participants:**

A county-level difference-in-differences study was conducted among adults aged 18 to 65 years in 112 manufacturing counties located in 30 commuting zones (primarily in the US South and Midwest) with at least 1 operational automotive assembly plant as of 1999. The study analyzed county-level changes from January 1, 1999, to December 31, 2016, in age-adjusted, county-level opioid overdose mortality rates before vs after automotive assembly plant closures in manufacturing counties affected by plant closures compared with changes in manufacturing counties unaffected by plant closures. Data analyses were performed between April 1, 2018, and July 20, 2019.

**Exposure:**

Closure of automotive assembly plants in the commuting zone of residence.

**Main Outcomes and Measures:**

The primary outcome was the county-level age-adjusted opioid overdose mortality rate. Secondary outcomes included the overall drug overdose mortality rate and prescription vs illicit drug overdose mortality rates.

**Results:**

During the study period, 29 manufacturing counties in 10 commuting zones were exposed to an automotive assembly plant closure, while 83 manufacturing counties in 20 commuting zones remained unexposed. Mean (SD) baseline opioid overdose rates per 100 000 were similar in exposed (0.9 [1.4]) and unexposed (1.0 [2.1]) counties. Automotive assembly plant closures were associated with statistically significant increases in opioid overdose mortality. Five years after a plant closure, mortality rates had increased by 8.6 opioid overdose deaths per 100 000 individuals (95% CI, 2.6-14.6; *P* = .006) in exposed counties compared with unexposed counties, an 85% increase relative to the mortality rate of 12 deaths per 100 000 observed in unexposed counties at the same time point. In analyses stratified by age, sex, and race/ethnicity, the largest increases in opioid overdose mortality were observed among non-Hispanic white men aged 18 to 34 years (20.1 deaths per 100 000; 95% CI, 8.8-31.3; *P* = .001) and aged 35 to 65 years (12.8 deaths per 100 000; 95% CI, 5.7-20.0; *P* = .001). We observed similar patterns of prescription vs illicit drug overdose mortality. Estimates for opioid overdose mortality in nonmanufacturing counties were not statistically significant.

**Conclusions and Relevance:**

From 1999 to 2016, automotive assembly plant closures were associated with increases in opioid overdose mortality. These findings highlight the potential importance of eroding economic opportunity as a factor in the US opioid overdose crisis.

## Key Points

**Question:**

Are closures of US automobile assembly plants associated with increases in opioid overdose mortality rates among working-age adults?

**Findings:**

In this difference-in-differences study, US manufacturing counties that experienced an automotive assembly plant closure were compared with counties in which automotive plants remained open from 1999 to 2016. Automotive assembly plant closures were associated with a statistically significant increase in county-level opioid overdose mortality rates among adults aged 18 to 65 years.

**Meaning:**

Automotive assembly plant closures were associated with increases in opioid overdose mortality, highlighting the potential importance of the role of declining economic opportunity in the US opioid overdose crisis.

## Teaser

This difference-in-differences study estimates the extent to which automotive assembly plant closures in the United States were associated with increasing opioid overdose mortality rates among working-age adults.

## Introduction

During the past 2 decades, the United States has experienced a widespread and significant increase in opioid overdose mortality, particularly among working-age adults.^1,2,3^ Recent work has shown that supply-side factors (eg, physician prescribing behavior and increasing availability of synthetic opioids) are associated with increased opioid dependence and risk of overdose.^4,5,6,7^ Consequently, policy and programmatic responses to the opioid overdose crisis have emphasized reducing the supply of opioids.^8,9,10,11,12^

The coincident increase in opioid overdose mortality during a time of worsening economic opportunity has also sparked interest in understanding the growing demand for opioids.^13,14^ Most prominently, Case and Deaton^15,16^ have argued that the erosion of longstanding economic opportunities has played a leading role in precipitating deaths from drug overdose, suicide, and other “deaths of despair.”^16^^(p398)^ However, studies examining the associations between unemployment, income, and opioid overdose mortality have yielded mixed findings.^17,18,19,20^ This lack of consensus may reflect the fact that standard economic measures do not adequately capture the fundamental and sustained decline in economic opportunity or the adverse socioeconomic and cultural climate that follows.^16,21,22,23,24^ Consistent with this hypothesis, studies based on other economic measures (eg, changes in employment opportunities owing to changes in international trade policy) have estimated strong associations with drug overdose mortality.^23,24,25^

To reconcile the mixed findings in the literature, we conducted a study to estimate the association between automotive assembly plant closures and opioid overdose mortality among working-age adults. We focused specifically on automotive assembly plant closures because they are often unexpected (to workers^26^), discrete, and both culturally and economically significant events, thus providing a unique opportunity to estimate the potential consequences of an acute, sustained decline in economic opportunities. Moreover, automotive plant closures have long been viewed as exemplars of the broader, gradual decline in US manufacturing that has occurred during the last 2 decades, a trend that has specifically been associated with the opioid overdose crisis.^16^ We compared changes in age-adjusted opioid overdose mortality rates among working-age adults living in manufacturing counties before vs after automotive assembly plant closures occurred compared with similar changes in manufacturing counties where plant closures did not occur.

## Methods

### Study Sample and Assignment of Exposure

The study period spanned from January 1, 1999, to December 31, 2016, corresponding to the full set of dates for which *International Statistical Classification of Diseases and Related Health Problems, Tenth Revision* (*ICD-10*)–coded death certificate data were available at the time of study design. Following the approach used in the academic literature and federal government reports,^27,28,29^ we built a database of all automotive assembly plants in operation as of 1999 using data from industry trade publications, automotive company websites, and newspaper articles. We identified the location of each plant and their dates of closure, if any (section 1 of the eAppendix in the Supplement provides further details; eTable 1 in the Supplement lists all identified plants.) Per University of Pennsylvania policy, institutional review board review was not required for this study given its use of deidentified data on deceased persons.

We next identified counties located within commuting zones that contained 1 or more automotive assembly plants. Counties were defined as exposed if, during the study period, there was a plant closure in the commuting zone in which they were located. Counties were defined as unexposed if their associated commuting zones experienced no closures of automotive assembly plants during the study period. We used commuting zones, which are contiguous groups of counties that are used to define local labor markets,^30^ to define exposure because individuals may not necessarily reside in the same county in which they work.^23,31^ In the 4 cases in which more than 1 automotive plant closure occurred within the commuting zone, exposure was assigned based on the date of the first closure.

The study sample was limited to manufacturing counties, defined as those in the top quintile nationwide with respect to the share of workers employed in manufacturing (eFigure 1 in the Supplement). This sample restriction focused our analysis on the manufacturing-dominated areas of the country^32,33^ most likely to be affected by automotive assembly plant closures and their downstream consequences. Our approach here follows the literature in using area-level measures to identify regions of the country at greatest risk,^34^ necessitated by the fact that death certificates lack information on the occupation of the deceased. Further details of the sample and exposure assignment are provided in section 2 of the eAppendix in the Supplement.

### Outcomes

The primary outcome was the county-level age-adjusted opioid overdose mortality rate among adults aged 18 to 65 years. We computed these rates by county of residence (the smallest available geographic identifier) and by calendar year using individual-level death certificate data from the US National Center for Health Statistics and population estimates from the US Census Bureau.^35,36^ Opioid overdose mortality rates were identified using *ICD-10* underlying cause codes X40 to X44, X60 to X64, X85, and Y10 to Y14 to identify drug overdose deaths and contributing cause codes T40.0 to T40.4 to identify deaths specific to opioid overdoses.^2^

To address possible bias from differential underreporting of opioid overdose deaths, we investigated the age-adjusted overall drug overdose mortality rate as a secondary outcome (section 3 of the eAppendix in the Supplement).^37,38^ We also separately examined prescription opioid (*ICD-10* codes T40.2, T40.3, and T40.4) and illicit opioid (opium and heroin [*ICD-10* codes T40.0 and T40.1]) overdose mortality rates as secondary outcomes.^39^

### Statistical Analysis

We first compared the socioeconomic characteristics of exposed and unexposed manufacturing counties using data from the 2000 Decennial Census (percentage of working-age adults, percentage of non-Hispanic white individuals, the unemployment rate, percentage of adults with a college degree, median household income, and the percentage of households below the federal poverty line). We used graphical methods to visualize unadjusted trends in the primary outcome of opioid overdose mortality rates per 100 000 adults aged 18 to 65 years separately within exposed and unexposed counties. We plotted these trends by event time (assigning the median year of plant closure, 2005, as the event year for unexposed counties).

We then estimated the association between automotive assembly plant closures and age-adjusted mortality rates at the county level using a difference-in-differences approach that allowed for the associations between exposure and outcome to vary over time (also known in the economics literature as an event study specification).^40,41,42^ Specifically, we estimated multivariable regression models in which the primary independent variables of interest were a series of binary indicators denoting each year before vs after automotive assembly plant closures. Unexposed manufacturing counties were assigned zeros for each of these indicator terms. This approach effectively compared changes in mortality rates in each yearly increment before vs after plant closures in manufacturing counties located in commuting zones in which a plant closure occurred against changes in mortality rates in manufacturing counties located in commuting zones in which no plant closure occurred. Unlike conventional applications of the difference-in-differences method, this specification is less prone to bias when the association between exposure and outcome changes over time.^42^ All regression models included county fixed effects, to adjust for potential confounding from time-invariant county-level factors (eg, rurality) or baseline differences in socioeconomic characteristics, and calendar year fixed effects, to adjust for nationwide secular trends in the outcomes, including supply-side factors (eg, national changes in opioid availability) or macroeconomic conditions (eg, the Great Recession). We did not adjust for standard county-varying and time-varying covariates, such as unemployment rates, poverty rates, or social capital, given that these are potential mediators of the association between automotive assembly plant closures and opioid overdose mortality rates. Adjusting for these variables would thus amount to overadjustment.^43^ Further details, including the estimation equation, are provided in section 4 of the eAppendix in the Supplement.

The key causal identifying assumption in difference-in-differences models is that outcomes in exposed counties would have continued along their same trajectories in the absence of exposure.^44^ This assumption cannot be directly tested, but potential violations can be probed by examining outcome trends for event years prior to plant closures. We expected the parallel trends assumption to be met in our study, given that plant closures were often announced to local communities with little advance notice, were rapidly implemented (ie, typically within 1-2 years) after announcement, and were unrelated to the productivity of the plant being closed (which is potentially associated with health status).^26^

The primary analysis focused on age-adjusted opioid overdose mortality rates among working-age adults (18-65 years). We also conducted analyses for subgroups based on age (18-34 vs 35-65 years), sex (men vs women), and race/ethnicity (non-Hispanic white vs all other racial/ethnic groups), given their differential exposures to the opioid overdose crisis.^2^ For all models, we computed 95% CIs adjusted for clustering within commuting zones, the geographical level at which exposure occurred.^45^ Observations were weighted by the 1999 county population size of working-age adults. All analyses were conducted using Stata/MP software, version 15.0 (StataCorp). All *P* values were from 2-sided tests and results were deemed statistically significant at *P* < .05. Data analyses were performed between April 1, 2018, and July 20, 2019.

### Sensitivity Analyses

We conducted several sensitivity analyses, which are described in greater detail in section 5 of eAppendix in the Supplement. We examined the sensitivity of our findings to modeling counts of opioid overdose deaths instead of mortality rates, using a generalized linear model method that allows for the estimation of relative changes in mortality,^46^ expanding the study sample to include commuting zones with automotive assembly plants that were excluded in the primary analysis, calculating SEs using a method that is more robust to smaller numbers of clusters, and using a different control group (to address potential spillovers between exposed and unexposed counties). We also reproduced the analysis under conditions in which it was unlikely to demonstrate the same findings: namely, estimating the association between plant closures and mortality outcomes in nonmanufacturing counties (defined as those in the bottom 4 quintiles nationwide with respect to the share of workers employed in manufacturing). Nonmanufacturing counties are less likely to be affected by automotive assembly plant closures, given that they are less likely to be composed of workers who are either employed or seek to be employed in the automotive industry. Finally, we assessed the extent to which our findings may be associated with selective migration out of exposed counties.

## Results

### Study Sample

**Figure 1.**
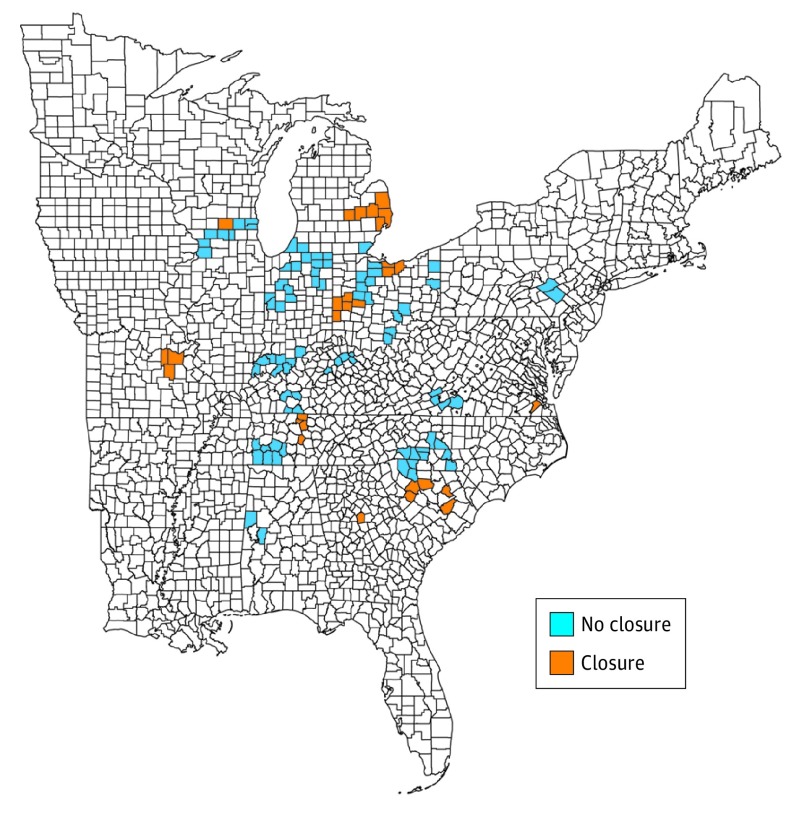
Sample Counties and Geographic Distribution of Automotive Assembly Plant Closures The 112 manufacturing counties that comprised the study sample were defined as those in which the percentages of employed residents working in manufacturing are in the top quintile nationwide. The 29 exposed manufacturing counties (Closure) were located in the 10 commuting zones in which an automotive assembly plant closure occurred between 1999 and 2016. The 83 unexposed manufacturing counties (No closure) were located in the 20 commuting zones in which automotive assembly plants in operation as of 1999 remained open throughout the duration of the study period.

Our study sample consisted of 112 counties situated in 30 commuting zones, which were primarily distributed across the midwestern and southern United States (Figure 1). These 112 counties accounted for 2.7% of the total population aged 18 to 65 years in the United States at baseline (1999) and 3.4% of the total number of deaths from opioid overdose nationwide among this age group during the study period (1999-2016).

Of the sample counties, 29 were exposed and were located in 10 commuting zones that experienced an automotive assembly plant closure during the study period. The remaining 83 counties were unexposed because they were located in 20 commuting zones that did not experience an automotive assembly plant closure. With 18 years of follow-up, our sample included 2016 county-year observations.

Baseline opioid overdose mortality rates and demographic and economic characteristics were similar in exposed vs unexposed counties (Table). Among the sample counties, plant closures occurred during the period from 2002 to 2009 (eFigure 2 in the Supplement).

**Table.** Baseline Characteristics of Counties Included in the Estimation Sample, Stratified by Exposure Status^a^

Baseline Characteristics	Mean (SD) Value	
	Closure	No Closure
Opioid overdose mortality rate (per 100 000 adults aged 18-65 y)	0.9 (1.4)	1.0 (2.1)
Working-age adults (18-65 y) in the county population, %	60.6 (1.4)	61.0 (1.5)
Non-Hispanic white adults in the county population, %	84.9 (18.8)	91.1 (10.7)
County-level unemployment rate, %	3.2 (0.8)	3.2 (0.8)
Adults in the county population who completed college, %	12.3 (3.2)	12.5 (3.7)
Household income (median, 2000), $^b^	45 977 (7513)	44 893 (6781)
Households in the county below 100% of the federal poverty line, %	9.6 (4.1)	9.3 (3.7)
No. of counties	29	83

^a^ Opioid overdose mortality rates are based on data from 2001, the year immediately preceding the first automotive plant closure in the sample. All other county-level variables are derived from the 2000 US Census, calculated as the percentage of individuals aged 16 years or older.

^b^ Median value within the county (county-level median), but the mean and the SD refer to the means and variation of median income levels across sample counties.

### Automotive Assembly Plant Closures and Drug Overdose Mortality

**Figure 2.**
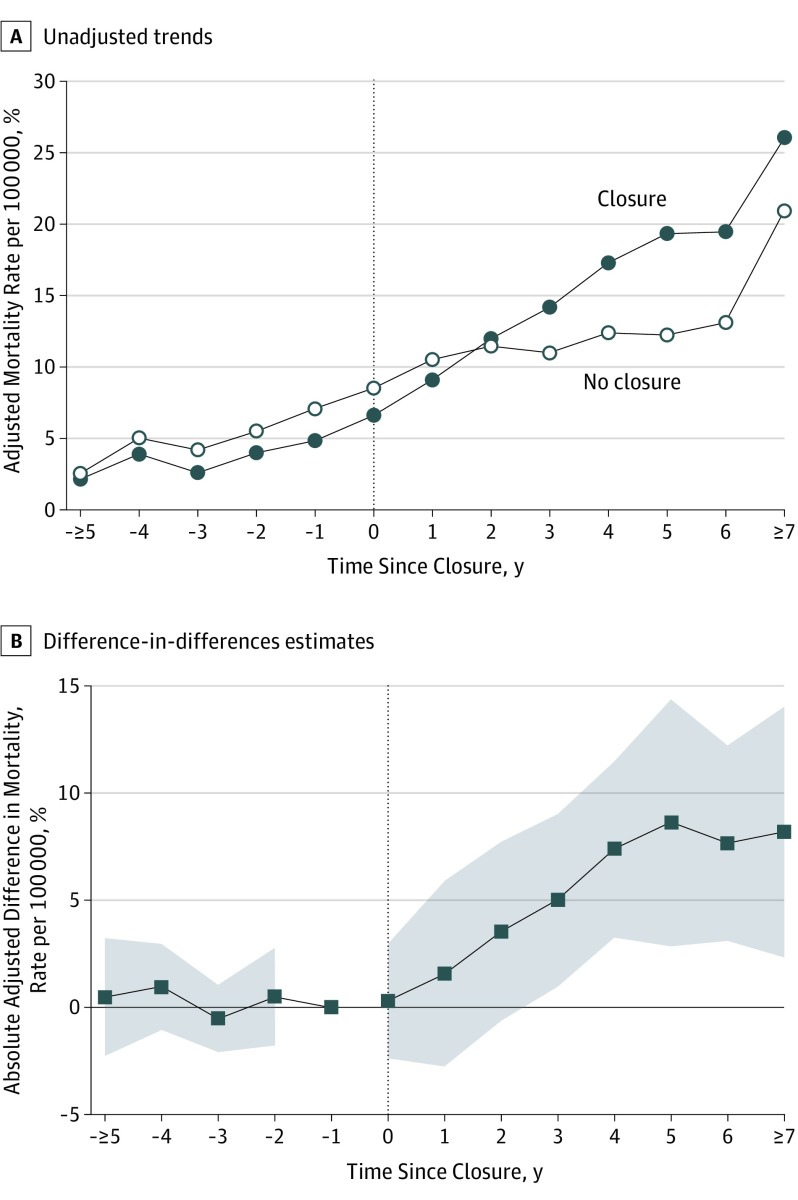
Unadjusted Trends and Adjusted Difference-in-Differences Estimates of the Association Between Automotive Assembly Plant Closures and Opioid Overdose Mortality Rates A, Unadjusted trends in county-level age-adjusted opioid overdose mortality rates among adults aged 18 to 65 years, separately for counties exposed and unexposed to automotive assembly plant closures. B, Adjusted difference-in-differences estimates (ie, the absolute adjusted difference between exposed and unexposed counties) for the same outcome (with the shaded areas representing 95% CIs) are plotted. In both panels, the x-axis represents the number of years relative to a plant closure, with event years 5 years or more years before exposure and 7 years or more years after combined into a single time point. The sample consisted of 2016 county-year observations, representing 29 exposed and 83 unexposed counties in 30 commuting zones followed from 1999 to 2016.

Unadjusted trends in opioid overdose mortality by time since event are provided in Figure 2A. Prior to plant closures, baseline opioid overdose mortality rates in exposed counties were lower than those in unexposed counties, with no evidence of differential trends in the primary outcomes. After plant closures, exposed counties experienced a greater increase in opioid overdose mortality rates compared with unexposed counties. Two years after plant closures, mortality rates were higher in exposed counties.

The adjusted difference-in-differences estimates of the association between automotive assembly plant closures and opioid overdose mortality displayed a similar pattern (Figure 2A). Each point on the y-axis reflects the difference in opioid mortality rates between exposed and unexposed counties relative to the year before plant closure (denoted as event time −1). Opioid overdose mortality increased in each of the first 5 years after plant closure and plateaued thereafter. Five years after exposure, mortality rates had increased by 8.6 deaths per 100 000 in exposed vs unexposed counties (95% CI, 2.6-14.6; *P* = .006). This estimate represents an 85% increase relative to the mortality rate of 12 deaths per 100 000 observed in unexposed counties at the same time point (Figure 2A). The pattern and magnitude of estimates were similar 5 years after exposure for the secondary outcome of overall drug overdose mortality (9.5 excess deaths per 100 000; 95% CI, 4.8-14.1; *P* < .001) (eFigure 3 in the Supplement), suggesting that differential identification and reporting of opioid deaths across exposed and unexposed counties did not substantively bias our findings.

**Figure 3.**
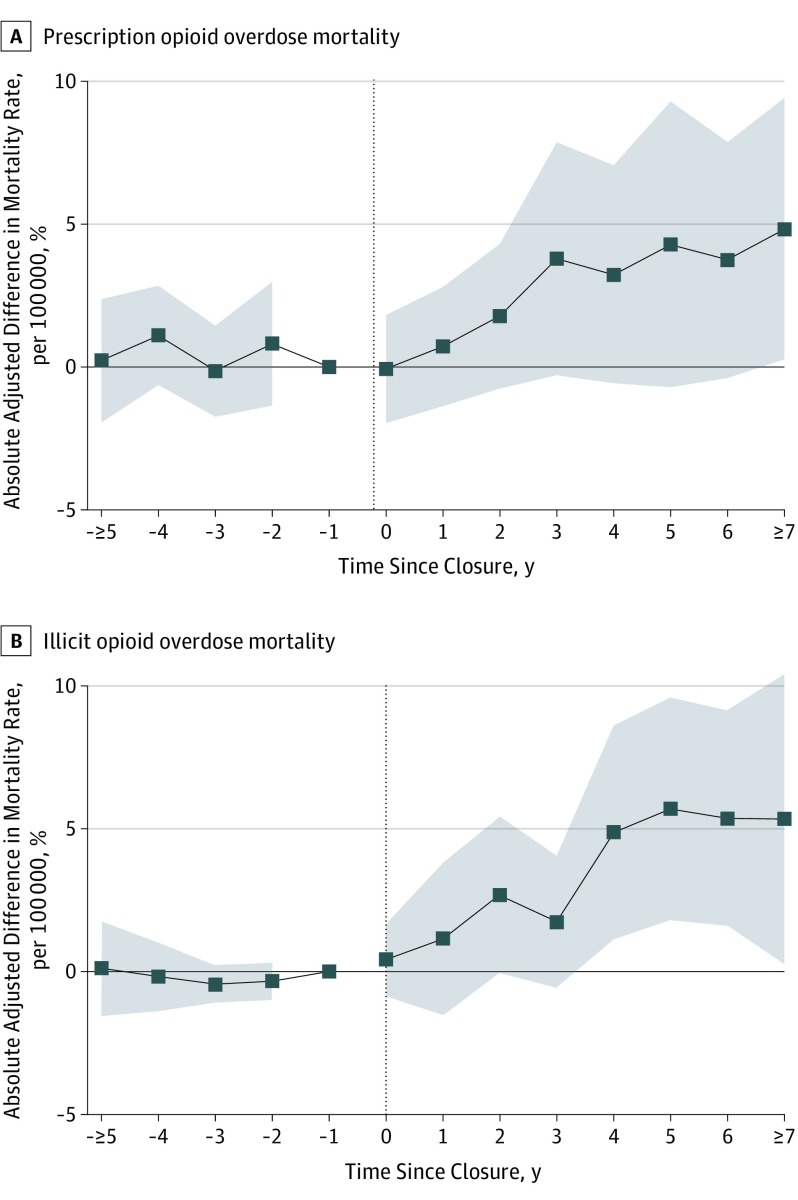
Difference-in-Differences Estimates of the Association Between Automotive Assembly Plant Closures and Prescription Opioid Overdose Mortality and Illicit Opioid Overdose Mortality A, Prescription opioid overdose mortality. B, Illicit opioid overdose mortality. Models are identical to those presented in Figure 2B, except here the dependent variables are opioid overdose mortality per 100 000 individuals aged 18 to 65 years from prescription opioids and illicit opioids. See Figure 2 caption for further details.

We found a similar pattern of results when we examined prescription vs illicit opioid overdose mortality separately (Figure 3). Five years after exposure, prescription opioid overdose mortality (Figure 3A) had increased by 4.4 deaths per 100 000 (95% CI, −0.8 to 9.6; *P* = .10), although this estimate was not statistically significant. Similarly, illicit opioid overdose mortality (Figure 3B) increased by 5.8 deaths per 100 000 (95% CI, 1.7-9.8; *P* = .001).

**Figure 4.**
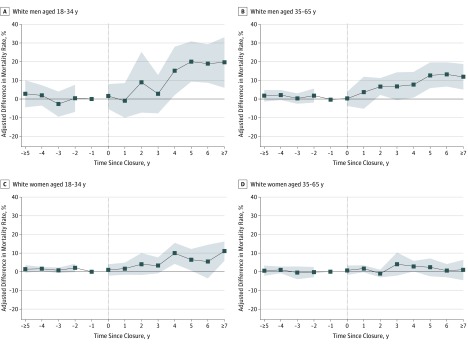
Difference-in-Difference Estimates for Opioid Overdose Mortality for Non-Hispanic White Adults, Stratified by Sex-Age Subgroups A, White men aged 18 to 34 years. B, White men aged 35 to 65 years. C, White women aged 18 to 34 years. D, White women aged 35 to 65 years. Models are identical to those in Figure 2B except here the dependent variable is opioid overdose mortality for each listed sex-age subgroup among non-Hispanic white adults. See Figure 2 caption for further details.

The magnitude of the association between plant closure and opioid overdose mortality was largest for non-Hispanic white men (Figure 4). Non-Hispanic white men aged 18 to 34 years experienced a relative increase of 20.1 deaths per 100 000 in exposed vs unexposed counties 5 years after a plant closure (95% CI, 8.8-31.3; *P* = .001). Similarly, non-Hispanic white men aged 35 to 65 years experienced a relative increase of 12.8 deaths per 100 000 (95% CI, 5.7-20.0; *P* = .001). Non-Hispanic white women aged 18 to 34 years experienced a relative increase in opioid overdose mortality of 6.4 deaths per 100 000 (95% CI, 0.4-12.3; *P* = .04), while the estimated association for older non-Hispanic white women (35-65 years) was smaller in magnitude and not statistically significant. Estimates for nonwhite men and women were generally smaller in magnitude, although we could not exclude clinically meaningful associations owing to the smaller population sizes of these subgroups (eFigure 4 in the Supplement). Among younger non-Hispanic white men and women, the estimates implied larger increases in mortality from illicit opioid overdoses vs prescription opioid overdoses, while the opposite pattern was found for older non-Hispanic white men (eFigure 5 in the Supplement).

### Sensitivity Analyses

The substantive findings remained unchanged when we modeled death counts instead of rates (eFigure 6 in the Supplement), used alternative methods to compute SEs (eTable 2 in the Supplement), expanded the study sample to include all commuting zones (eFigure 7 in the Supplement), and used an alternative control group (eFigure 8 in the Supplement). When we restricted the sample to nonmanufacturing counties with the intent of reproducing the analysis under conditions unlikely to generate the same findings, the estimated association between automotive plant closure and opioid overdose mortality was substantially smaller in magnitude and not statistically significant (eFigure 9 in the Supplement). We found no evidence of a substantively or statistically significant association between plant closures and changes in migration rates, which suggests that our findings were not driven by differential outmigration from counties experiencing plant closures (eFigure 10 in the Supplement).

## Discussion

In this difference-in-differences study of 112 US manufacturing counties, we found that automotive assembly plant closures were associated with increased mortality from opioid overdose. The estimated association was consistent with temporal progression and was specific to manufacturing counties. The estimates imply that, 5 years after a plant closure, opioid overdose mortality rates were 85% higher, in relative terms, than what would have been expected had exposed counties followed the same outcome trends as unexposed counties. The burden of this increase in opioid overdose mortality was primarily borne by non-Hispanic white men.

Our findings illustrate the importance of declining economic opportunity as an underlying factor associated with the opioid overdose crisis. In particular, our findings, combined with a growing body of research demonstrating adverse associations between trade-related industrial decline and drug overdose mortality,^23,24^ lend support to the view that the current opioid overdose crisis may be associated in part with the same structural changes to the US economy that have been responsible for worsening overall mortality among less-educated adults since the 1980s.^47,48^ Declining economic opportunity is one hypothesized mechanism associated with these longer-term trends.^15,16,21,49^ Given our study context, this argument is most relevant for worsening population health trends in the midwestern and southern United States, regions that have experienced some of the largest increases in opioid overdose mortality^50,51^ and in which the automotive industry has long been economically and culturally significant. In addition, our focus on an acute, sustained decline in economic opportunity may help reconcile prior disparate findings about the importance of the economic factors associated with the opioid overdose crisis, which are based primarily on standard measures of economic status (eg, unemployment and per capita income).^17,18,19,20^

Our findings should not be interpreted in such a way as to diminish the role of opioid supply, either from physician prescriptions or from illicitly made and supplied synthetic substances, in the US opioid overdose crisis. Instead, the findings suggest that successful approaches to address the opioid overdose crisis will likely involve complementary interventions to reduce the prescription and illicit opioid supply as well as interventions to diagnose and treat substance use disorders in regions of the country hardest hit by structural economic change. The development of a national resilience strategy, which includes heightened screening and surveillance and the development of scalable community-based interventions,^52^ educating and empowering clinicians to identify and address structural forces that may shape patient health,^53^ and increasing engagement of community agencies and health care systems in addressing key social determinants of health, could be important in mitigating the negative health consequences of economic shocks.^54,55^ In addition, social policies to mitigate growing disparities in economic opportunity will also be required,^13^ particularly as economic opportunities in sectors such as manufacturing—where jobs are prone to be automated away or offshored—are likely to continue to decline for the foreseeable future.^56^

### Limitations

Interpretation of our findings is subject to several limitations. First, despite the robustness of the findings to several sensitivity analyses, we could not definitively rule out the possibility that the estimated associations could be explained by residual confounding. There could be unmeasured time-varying factors at the level of the county coincident with these unexpected plant closures, above and beyond national secular trends, that could also be associated with changes in the outcomes. Second, owing to the inherent limitations of vital statistics records, we relied on a proxy measure for exposure assignment. It is therefore possible that measurement error could have attenuated the magnitude of our estimates.

Third, our findings may not generalize beyond this specific study context. In particular, the sample counties accounted for only a small share (2.7%) of the US adult population at the beginning of the study period, and automotive assembly plant closures represent a unique, albeit large-scale, shock in 1 specific industry. However, the study regions share similarities with the temporal and demographic patterns in opioid overdose mortality observed nationwide, and other manufacturing industries have also experienced similar trajectories of decline as the automotive industry. Future work could apply the difference-in-differences approach used in this study to examine the population health consequences of declining employment opportunities in the manufacturing sector. Future research could also extend our findings to mortality from other causes of death tied to despair, such as alcoholic liver disease and suicide,^15^ as well as other causes of death for which mortality rates have stagnated or increased in recent years, such as cardiometabolic diseases.^57^

Fourth, we were unable to definitively elaborate the mechanisms underlying our results. Although job loss is likely an important factor,^41,58^ other important mediators could include broad changes in expectations about future economic mobility and the social and cultural change that follows the death of historically and culturally significant industries.^16,23,59^ Fifth, our study was not powered to identify moderators of the association between automotive plant closures and opioid overdose deaths. Prior prevalence of prescription opioids, baseline social capital, or preceding economic conditions and policies may all play a role in diminishing resilience to declining economic fortunes.^16^ Identifying key mechanisms underlying our findings and the factors that moderate the association between economic opportunities and opioid overdose mortality remain important areas for future research.

## Conclusions

From 1999 to 2016, automotive assembly plant closures were associated with increased county-level opioid overdose mortality. These findings highlight the potential importance of declining economic opportunity as a factor associated with the US opioid overdose crisis.

## Notes

Supplement. **eAppendix.** Materials and Methods **eTable 1.** Automotive Assembly Plants in Operation as of 1999, Location, and Closure Dates **eTable 2.** Event Study Estimates From Figure 2 of Main Text, Including Alternate Calculation of Standard Errors **eFigure 1.** US Counties by Quintile of the Manufacturing Share of Workforce in 2000 **eFigure 2.** Trends in Share of Sample Manufacturing Counties Exposed to Commuting Zone Automotive Assembly Plant Closure **eFigure 3.** Adjusted Difference-in-Differences Estimates for Overall Drug Overdose Mortality **eFigure 4.** Adjusted Difference-in-Differences Estimates for Nonwhites, Stratified by Sex-Age Subgroups **eFigure 5.** Adjusted Difference-in-Differences Estimates by Class of Opioid Stratified by Demographic Subgroup **eFigure 6.** Adjusted Difference-in-Differences Estimates, Modelling Overdose Deaths as a Count Variable **eFigure 7.** Adjusted Difference-in-Differences Estimates for an Expanded Sample That Includes Manufacturing Counties From Each Commuting Zone Excluded From Main Sample **eFigure 8.** Adjusted Difference-in-Differences Estimates Using an Alternate Control Group **eFigure 9.** Adjusted Difference-in-Differences Estimates in Nonmanufacturing Counties **eFigure 10.** Differences in Net Migration Rates by Exposure to Automotive Assembly Plant Closures
