# Supplementary material for: Association Between Automotive Assembly Plant Closures and Opioid Overdose Mortality in the United States: A Difference-in-Differences Analysis
Source: JAMA Intern Med. 2019 Dec 30;180(2):254–62. doi: 10.1001/jamainternmed.2019.5686 (PMC6990761; doi:10.1001/jamainternmed.2019.5686)
Supplement: Supplement. — eAppendix. Materials and Methods eTable 1. Automotive Assembly Plants in Operation as of 1999, Location, and Closure Dates eTable 2. Event Study Estimates From Figure 2 of Main Text, Including Alternate Calculation of Standard Errors eFigure 1. US Counties by Quintile of the Manufacturing Share of Workforce in 2000 eFigure 2. Trends in Share of Sample Manufacturing Counties Exposed to Commuting Zone Automotive Assembly Plant Closure eFigure 3. Adjusted Difference-in-Differences Estimates for Overall Drug Overdose Mortality eFigure 4. Adjusted Difference-in-Differences Estimates for Nonwhites, Stratified by Sex-Age Subgroups eFigure 5. Adjusted Difference-in-Differences Estimates by Class of Opioid Stratified by Demographic Subgroup eFigure 6. Adjusted Difference-in-Differences Estimates, Modelling Overdose Deaths as a Count Variable eFigure 7. Adjusted Difference-in-Differences Estimates for an Expanded Sample That Includes Manufacturing Counties From Each Commuting Zone Excluded From Main Sample eFigure 8. Adjusted Difference-in-Differences Estimates Using an Alternate Control Group eFigure 9. Adjusted Difference-in-Differences Estimates in Nonmanufacturing Counties eFigure 10. Differences in Net Migration Rates by Exposure to Automotive Assembly Plant Closures [file jamainternmed-180-254-s001.pdf]

Supplementary Online Content

Venkataramani AS, Bair EF, O'Brien RL, Tsai AC. Association between automotive assembly plant closures and opioid overdose mortality in the United States: a difference-in-differences analysis. *JAMA Intern Med.* Published online December 30, 2019. doi:10.1001/jamainternmed.2019.5686

eAppendix. Materials and Methods
eTable 1. Automotive Assembly Plants in Operation as of 1999, Location, and Closure Dates
eTable 2. Event Study Estimates From Figure 2 of Main Text, Including Alternate Calculation of Standard Errors
eFigure 1. US Counties by Quintile of the Manufacturing Share of Workforce in 2000
eFigure 2. Trends in Share of Sample Manufacturing Counties Exposed to Commuting Zone Automotive Assembly Plant Closure
eFigure 3. Adjusted Difference-in-Differences Estimates for Overall Drug Overdose Mortality
eFigure 4. Adjusted Difference-in-Differences Estimates for Nonwhites, Stratified by Sex-Age Subgroups
eFigure 5. Adjusted Difference-in-Differences Estimates by Class of Opioid Stratified by Demographic Subgroup
eFigure 6. Adjusted Difference-in-Differences Estimates, Modelling Overdose Deaths as a Count Variable
eFigure 7. Adjusted Difference-in-Differences Estimates for an Expanded Sample That Includes Manufacturing Counties From Each Commuting Zone Excluded From Main Sample
eFigure 8. Adjusted Difference-in-Differences Estimates Using an Alternate Control Group
eFigure 9. Adjusted Difference-in-Differences Estimates in Nonmanufacturing Counties
eFigure 10. Differences in Net Migration Rates by Exposure to Automotive Assembly Plant Closures

This supplementary material has been provided by the authors to give readers additional information about their work.

© 2019 Venkataramani AS et al. *JAMA Internal Medicine*.

eAppendix. Materials and Methods

Section 1. Identification of automotive assembly plants

Our strategy to identify automotive assembly plant closures followed the approach used in the academic literature and federal government reports, which involves triangulating data from industry trade publications, automotive company websites, and newspaper articles.[1-3] We first obtained an initial listing of automotive assembly plants in operation in North America as of 2005-2006 from Automotive News (AutoNews.com),[4] a leading industry trade publication. This was the earliest publicly-available and industry-verified census of automotive assembly plants during our study period. We considered all plants that were involved in manufacturing of automobiles, trucks, and heavy trucks. To identify plants that were in operation as of 1999, but had closed by 2005-2006, we conducted a series of Google and Google News searches using the each of the following search terms:

[Company name] [Year] plant
[Company name] [Year] auto plant
[Company name] [Year] assembly
[Company name] [Year] assembly plant
[Company name] [Year] auto assembly
[Company name] [Year] auto assembly plant
[Company name] [Year] plant closed
[Company name] [Year] plant closure
[Company name] [Year] auto plant closed
[Company name] [Year] auto plant closure
[Company name] [Year] assembly closed
[Company name] [Year] assembly closure
[Company name] [Year] assembly plant closed
[Company name] [Year] assembly plant closure
[Company name] [Year] auto assembly closed
[Company name] [Year] auto assembly closure
[Company name] [Year] auto assembly plant closed
[Company name] [Year] auto assembly plant closure

The field [Company name] refers to each of the following automotive manufacturers: AM General, Autoalliance, BMW, FCA US LLC (or "DaimlerChrysler" or "Chrysler"), Ford, Freightliner, General Motors (or "GM" or "G.M"), Honda, Hyundai, International, Kenworth, Mack, Mitsubishi, Nissan, Nummi, Peterbilt, Sterling, Subaru, Toyota, Volvo, and Western Star. The field [Year] refers to individual years between 1999 and 2005. Using this strategy, we found six additional automotive assembly plants that were operational as of 1999 but closed before 2005.

To identify automobile assembly plant closures from 2005 onwards, we used a combination of company websites (e.g. https://corporate.ford.com/company/operation-list.html#s1f0) and Google news searches using either specific plant names or company names with plant locations. Specifically, we conducted searches using the following terms:

[Plant name] closed
[Plant name] closure
[Plant name] plant closed
[Plant name] plant closure

© 2019 Venkataramani AS et al. *JAMA Internal Medicine*.

[Company name] [Plant location city], [Plant location state]
[Company name] [Plant location city], [Plant location state] auto plant
[Company name] [Plant location city], [Plant location state] auto plant closed
[Company name] [Plant location city], [Plant location state] auto plant closure
[Company name] [Plant location city], [Plant location state] auto assembly
[Company name] [Plant location city], [Plant location state] auto assembly closed
[Company name] [Plant location city], [Plant location state] auto assembly closure

Where [Plant name], [Company name], [Plant location city], and [Plant location state] are fields specific to each individual plant that we identified as being in operation as of 1999. We cross-checked each potential closure and closure date using additional Google searches.

The list of automotive assembly plants identified through our search algorithm, along with their location, and, if relevant, year of closure are provided eTable 1. The list is divided by plants that were represented in the main study sample versus those that were not, based on the sample restrictions discussed in Section 2 of this Supplement.

Section 2. Sample and exposure

To define the study sample, we first restricted the study sample to all counties within commuting zones that contained at least one operational automotive assembly plant as of 1999. Commuting zones represent groups of counties that capture predominant residential and commuting patterns. The use of commuting zones to define exposure therefore accounts for the possibility that individuals likely to be affected by automotive assembly plant closures may live in a county other than the one in which the plant was located. Commuting zones have been widely used to define local labor markets.[11-13] Because our study period ranged from 1999-2016, we used commuting zone definitions from the year 2000. Counties were considered exposed to an automotive assembly plant closure if they were located in a commuting zone that experienced a plant closure during the study period. In the 4 commuting zones where more than one automotive plant closure occurred, we assigned exposure based on the year of the first closure.

We defined counties that were most likely to be affected by automobile assembly plant closures using data on the share of county residents employed in manufacturing at the beginning of the study period. Specifically, we restricted our sample to counties in the top quintile nationwide with respect to the share of employed residents aged 16 years and above who were working in the manufacturing sector, based on data from the 2000 Decennial Census[14] ("manufacturing counties," eFigure 1). The use of the top quintile versus bottom four quintiles of the share of the workforce employed in manufacturing to define manufacturing counties follows from prior work, which (1) demonstrated that top quintile manufacturing counties experienced distinct socioeconomic trends during the study period,[15] and (2) defined the U.S. manufacturing sector as consisting of ~600 counties (i.e., approximately 1/5th of all U.S. counties).[16] Imposing this sample restriction meant that 18 of the 48 commuting zones with at least one automotive assembly plant in operation as of 1999 were excluded, as they did not contain any counties with a share of manufacturing workers in the highest national quintile. As we discuss in Section 5C, our substantive findings were unchanged if we employed alternate definitions of exposure that included counties from all 48 of these commuting zones.

© 2019 Venkataramani AS et al. *JAMA Internal Medicine*.

Our approach to identify sample counties was motivated in part by limitations in vital statistics and employment record data. Vital statistics data do not include information on previous occupation or industry of employment, and data on relevant proxies such as education are often missing or measured with error.[17] Employment databases, such as the U.S. Census Bureau's Quarterly Workforce Indicators,[18] allow for an exact enumeration of individuals employed in the automotive industry. However, the assignment of counties in these data are based on place of employment and not place of residence, which creates similar potential for measurement error as in the vital statistics data (which identify only the county of residence or death).

Our approach was also motivated by the fact that the consequences of an automotive assembly plant closure likely extend beyond just those workers employed in the automotive industry. These spillovers are likely present because individuals employed in other manufacturing industries have long sought employment in the historically higher-paying automotive sector.[19] Moreover, contraction of opportunities within the automotive sector likely affected other workers in other manufacturing industries through reduced demand for automotive parts from component suppliers[1,20] and potentially through changing expectations about the potential for socioeconomic mobility through manufacturing work.

Our final study sample consisted of 112 manufacturing counties situated in 30 commuting zones, including 29 exposed counties located in 10 commuting zones in which an automotive assembly plant closed during the study period, and 83 unexposed counties located in 20 commuting zones in which all automotive assembly plants remained open during the study period.

Section 3. Outcome variables

The primary outcome was the annual age-adjusted opioid overdose rate for 18-65-year-old adults in the county. We calculated these rates using restricted-access, individual-level multiple cause of death vital statistics data from the U.S. National Center for Health Statistics (NCHS) from January 1, 1999 to December 31, 2016.[5] We followed the strategy recommended by the U.S. Centers for Disease Control and Prevention by first using ICD-10 underlying cause codes X40-X44, X60-X64, X85, Y10-Y14 to identify drug overdose deaths and then using contributing cause codes T40.0-T40.4 to identify deaths specific to opioid overdoses.[6,7] Doing so, we identified a total of 10,759 opioid overdose deaths of 18-65 year-old adults in the 112 sample counties during the study period. We then used these data, along with population counts from the U.S. Census Bureau,[8] to compute age-adjusted rates per 100,000 individuals (based on a direct standardization method). Counties were assigned based on reported county of residence for each decedent in the NCHS data. We used a similar procedure to construct our secondary outcomes: rates of overall drug overdose mortality (ICD-10 codes X40-X44, X60-X64, X85, Y10-Y14), prescription opioid overdose mortality (subset of drug overdose deaths assigned contributing cause codes, T40.2, T40.3, and T40.4), and illicit opioid overdose mortality (subset of drug overdose death contributing cause codes, T40.0 and T40.1).[7]

We examined overall drug overdose mortality as a secondary outcome in order to address potential bias from underreporting of opioids as a specific cause of drug overdose mortality during the study period.[9] This potential bias includes differential changes in the likelihood of identifying opioids as the causal agent specifically related to plant closures (e.g., due to rising

© 2019 Venkataramani AS et al. *JAMA Internal Medicine*.

overall drug overdose mortality rates raising concerns among medical examiners). The possibility of such bias is suggested by geographic variation in the identification of different contributing causes of drug overdose deaths.[10] The concordant results for both opioid and overall drug overdose mortality (Figure 2) suggests that any bias from differential reporting was unlikely to have been substantial.

We used the same procedures to construct subgroup-specific opioid overdose mortality rates reflecting each combination of age (18-34 versus 35-65), sex (women versus men), and race/ethnicity (non-Hispanic white versus all other).

Section 4. Statistical model

For each primary and secondary outcome, we estimated the following least squares multivariable regression model:

$$(1)\quad Y_{ijt} = \alpha_0 + \sum_{p=-5}^{7} \alpha_p (Closure_{jp}) + \mu_i + \theta_t + \varepsilon_{ijt}$$

where *i* indexes the county, *j* the commuting zone within which the county is located, and *t* the calendar year. The subscript *p* refers to event years, which are annual intervals relative to the calendar year of automotive assembly plant closure. For example, period -3 refers to 3 years before plant closure. Event periods 5 or more years prior to plant closure were combined into a single bin, as were event periods 7 or more years after plant closure. This aggregation follows prior literature[21] as a means of avoiding difficulties in interpreting coefficients due to sample size imbalances created by differences in the timing of plant closures. The variable $Closure_{jp}$ denotes a series of binary indicators = 1 if the closure of an automotive assembly plant in operation since 1999 had occurred in commuting zone *j* during or before the calendar year associated with event-time period *p*. Y_{ijt} denotes the outcome of interest. County fixed effects (denoted by μ_i) captured time-invariant differences in socioeconomic and cultural characteristics across sample counties. Nationwide secular trends in the outcomes, for example, due to the Great Recession or differential changes in availability of prescription or illicit opioids were captured by calendar year fixed effects (denoted by θ_t).

This model estimates the difference in outcomes for leads and lags of plant closure relative to a reference year (event time -1) and relative to all manufacturing counties in commuting zones where a plant had not closed during the study period (i.e., for whom $Closure_{jp}$ is equal to zero for all event periods). These relative differences are captured by the coefficients α_p, estimates for which are plotted in all of the main and supplement figures.

By allowing associations between exposure and the outcome to vary over time, our specification represents a generalization of the method of difference-in-differences - known as the "event study specification" in modern econometrics.[22-24] Specifically, in cases where the timing of exposure varies across units and where effects vary with time, event study models are preferred to standard difference-in-differences models because the single coefficient reported in difference-in-differences analyses represents a weighted average of all combinations of comparisons between sample units.[22] However, in some of these comparisons, units exposed earlier may serve as controls for units exposed later. If differences in outcomes are increasing

© 2019 Venkataramani AS et al. *JAMA Internal Medicine*.

with event time (as is the case for the present study), the estimates from these particular comparisons will be attenuated toward the null. Hence, the single difference-in-differences estimate may provide a misleading estimate of the true association between exposure and outcome. The event study specifications, which also provide a more transparent test of violations of the parallel trends assumption required for causal inference,[26] avoid this problem by indexing the reference point to time since the event (as opposed to calendar time) and by allowing associations to vary over time.

For all models, we computed 95% confidence intervals adjusting for serial correlation in the outcome at the commuting zone level.[27] We weighted all regressions by county population size at baseline (1999).

Section 5. Sensitivity analyses

A. Modelling overdose deaths as a count variable

We estimated our main regression model (Equation 1) using least squares given this estimator is less prone to finite sample bias from the inclusion of numerous fixed effects (a well-known problem that arises with maximum likelihood estimators such Poisson or negative binomial regression[28]). We nevertheless assessed the sensitivity of our findings to this choice by modelling the number of opioid overdose deaths, using a generalized linear model with a negative binomial distribution and a log-link (and with county-year population specified as the exposure).[29] The resulting coefficients can be interpreted as relative changes in mortality rates.

The substantive findings were robust to using this alternate estimate strategy (eFigure 6). In particular, the estimates suggest that at 5 years after a plant closure, opioid overdose mortality rates increased by 85% (95% CI: 41%, 128%, p<0.001) in exposed counties relative to unexposed counties. (The corresponding estimate from our main specification implies a 75% increase relative to unexposed counties by the same time point.)

B. Wild-cluster bootstrap-t procedure for variance estimation

The Huber-White method to account for geographic clustering may result in standard errors that are biased downwards in cases where there are few clusters.[30] What constitutes a small number of clusters depends on the application. Our main study sample specified 30 clusters (commuting zones), which is generally considered a large enough number of clusters according to conventional rules of thumb. Nevertheless, we tested the robustness of our statistical inference using the wild cluster bootstrap method.[31] To implement this procedure, we used the Stata command `-bootwildct-` developed by Bansi Malde and Molly Scott (available at https://www.ifs.org.uk/publications/6231). As shown in eTable 2, the *p*-values on the event study coefficients were substantively unchanged when using this alternate procedure.

C. Including counties from commuting zones excluded from main sample

As discussed in Section 2, we restricted our sample to counties in the top quintile nationwide with respect to the share of employed residents working in the manufacturing sector, in order to

© 2019 Venkataramani AS et al. *JAMA Internal Medicine*.

identify areas of the country most likely to be economically affected by automotive assembly plant closures. However, doing so excluded counties in 18 of the 48 commuting zones with at least one operational automotive assembly plant as of 1999. To assess whether our results were sensitive to the inclusion of these commuting zones, we added to our main sample the county in each excluded commuting zone with the highest share of residents working in the manufacturing industry. Results for this alternate sample, which included 130 counties (37 exposed and 93 unexposed), were similar to those for the main study sample (eFigure 7).

D. Alternate control group

Our primary regression models defined unexposed manufacturing counties as those located in commuting zones without an automotive assembly plant closure. We hypothesized that these unexposed counties were likely to follow similar pre-existing trends in the outcomes compared with exposed counties, given that they would also be subject to similar automotive industry-specific social, economic, and cultural factors that may be relevant to population health. However, plant closures in one commuting zone may cause automotive workers in commuting zones where plants have remained open to experience economic uncertainty, which may be consequential for health.[32] Another possibility is that automotive manufacturers preferentially closed plants in areas where their workers were experiencing downward trends in health. However, both the known rationales for specific plant closure decisions[19] and the absence of differential pre-existing trends in the outcome between exposed and unexposed counties (Figure 2) suggests that bias from selective plant closure is unlikely.

These potential spillovers could violate the Stable Unit Treatment Value Assumption (SUTVA).[26] To assess the robustness of our findings to this potential violation, we repeated the analysis with an alternate control group: manufacturing counties located in non-automotive commuting zones that were situated in the same states as commuting zones with automotive plant closures. The restriction of counties to the same states helps to ensure similar exposure to broad, regional trends in social or economic factors that could influence health outcomes. However, this control group should be less prone to cross-commuting zone spillovers (though the problem is not fully eliminated given that firms supplying automobile component parts, who would face the downstream consequences of plant closures, tend to locate in similar regions as automotive plants[1]). The estimates using this alternate control group were substantively similar to our main findings (eFigure 8), suggesting that cross-commuting zone spillovers are unlikely to bias our findings.

E. Repeating the analysis under conditions expected to produce a null result

We did not expect to find strong associations between automotive assembly plant closures and opioid overdose mortality in non-manufacturing counties (i.e., counties in the bottom four quintiles of the county workforce employed in manufacturing). Consistent with this expectation, the estimated associations between plant closure and opioid overdose mortality in non-manufacturing counties were smaller in magnitude and not statistically significant (eFigure 9).

© 2019 Venkataramani AS et al. *JAMA Internal Medicine*.

F. Selective migration

The association between automotive assembly plant closures and rising opioid overdose mortality rates may reflect, in part, selective outmigration of individuals at lower risk of developing substance use disorders. While recent work in labor economics suggests that differential outmigration after adverse economic events may be minimal,[12,33] we nevertheless examined whether manufacturing counties exposed to plant closures experienced greater (net) outmigration relative to unexposed manufacturing counties.

We obtained intercensal, county-level data on net migration rates from the University of Wisconsin-Madison, Net Migration Patterns for US Counties database.[34] We used these data to create net migration rates by each combination of age group (18-34 years versus 35-64 years) and sex. We investigated these groups separately given potential differences in the propensity to migrate, along with differential exposure to automotive assembly plant closures (Figure 3). Because these data are only available at intercensal (i.e., decadal) intervals, we could not estimate the event study specification in Equation 1. Instead, we estimated the following least squares regression model, for each age-sex group:

$$(2) \quad \Delta MigrationRate_{ij} = \beta_0 + \beta_1(Closure, 2005 - 2009_j) + \beta_2(Closure, 2006 - 2009_j) + \varepsilon_{ijt}$$

where *i* indexes the county and *j* the commuting zone within which the county is situated. The outcome variable $\Delta MigrationRate_{ij}$ represents the difference in decadal net migration rates (i.e., between the 2010 and 2000 censuses and between the 2000 and 1990 census). Positive values of this measure reflect increasing in-migration (or decreasing outmigration) while negative values reflect either increasing outmigration or slowing of in-migration. The terms $(Closure, 2002 - 2005_j)$ and $(Closure, 2006 - 2009_j)$ are binary indicators = 1 if the manufacturing county was located in a commuting zone where an automotive assembly plant in operation at the start of the study period closed during the specified time frame. We split closures to take into account any potential lagged effects of economic decline on migration.[35]

Equation 2 assesses whether net migration rates changed more in manufacturing counties located in a commuting zone where an automotive assembly plant closed versus manufacturing counties not exposed to plant closure . Because the dependent variable is the change in decadal net migration rates, and the independent variables effectively capture a commuting zone change in the operational status of one or more automotive assembly plants, Equation 2 effectively represents a first-differences model. Like our event study specifications, which include county-specific fixed effects, first-differences models adjust for potential confounding by time-invariant, county-specific variables, whether observed or unobserved.[36] This specification is the closest analog to the model in Equation 1 that was possible to fit given the constraints of the migration data.

Consistent with the recent labor economics literature,[12,33] we found no evidence that automotive assembly plant closures were associated with net migration rates for any era of plant closure (eFigure 10). Across specifications, even the estimates with the largest magnitudes represent only a 0.13 standard deviation decrease in net *in*-migration (not outmigration) rates.

© 2019 Venkataramani AS et al. *JAMA Internal Medicine*.

There are two caveats to these findings. First, the confidence intervals are relatively wide, so we cannot exclude the possibility that there were substantively meaningful differences in net out-migration rates attributable to plant closures. Second, the findings only apply to a time frame of 8 years post plant closure (because the last decadal time point to assess net migration was 2010 and the first closure in our sample was 2002). Thus, we cannot rule out differences in out-migration over a longer time frame. That said, the null findings here are useful to support the veracity of our event study estimates through at least 6 years of follow-up after plant closure.

Section 6. eReferences

1. Rubenstein JM. The evolving geography of production - is manufacturing activity moving out of the Midwest? Evidence from the auto industry? Federal Reserve Bank of Chicago working paper No SP-3 1996.
2. Ramey V, Vine D, Bresnahan TF. Weekly Production Scheduling at Assembly Plants in the United States Automobile Industry: 1972-1983 and 1990-2001. Ann Arbor, MI: Inter-university Consortium for Political and Social Research; 2009.
3. Platzer MD, Harrison GJ. The U.S. Automotive Industry: National and State Trends in Manufacturing Employment. Washington, D.C.: Congressional Research Service; 2009.
4. North America Car and Truck Assembly Plants 2005-2006: Automotive News; 2006. (Accessed December 3 2018, at https://www.autonews.com/assets/PDF/CA4984224.PDF)
5. National Center for Health Statistics, Centers for Disease Control and Prevention. NCHS data release and access policy for micro-data and compressed vital statistics files. (Accessed February 14 2019, at https://www.cdc.gov/nchs/nvss/dvs_data_release.htm.)
6. Scholl L, Seth P, Kariisa M, Wilson N, Baldwin GT. Drug and opioid-involved overdose deaths - United States, 2013-2017. Morbidity and Mortality Weekly Report 2019;67:1419-27.
7. Prescription Drug Overdose Team - Division of Unintentional Injury Prevention and Control. Prescription Drug Overdose Data and Statistics: Guide to ICD-9-CM and ICD-10 Codes Related to Poisoning and Pain. Atlanta, GA: U.S. Centers for Disease Control; 2013.
8. National Center for Health Statistics, Centers for Disease Control and Prevention. US census populations with bridged race categories. (Accessed February 14, 2019, at https://www.cdc.gov/nchs/nvss/bridged_race.htm.)
9. Ruhm CJ. Corrected US opioid-involved drug poisoning deaths and mortality rates, 1999–2015. Addiction 2018;113.
10. Dwyer-Lindgren L, Bertozzi-Villa A, Stubbs RW, et al. Trends and patterns of geographic variation in mortality from substance use disorders and intentional injuries among US counties, 1980-1984. JAMA Internal Medicine 2018;319:1013-23.
11. Autor D, Dorn D, Hanson G. When work disappears: manufacturing decline and the falling marriage-market value of men. American Economic Review: Insights, Forthcoming.
12. Autor D, Dorn D, Hanson G, Song J. Trade adjustment: worker level evidence. Quarterly Journal of Economics 2014;129:1799-860.
13. Chetty R, Hendren N, Kline P, Saez E. Where is the Land of Opportunity? The Geography of Intergenerational Mobility in the United States Quarterly Journal of Economics 2014;129:1553-623.
14. United States Census Bureau. Summary Files 1 and 3, Census 2000. Social Explorer.
15. Nunn R, Parsons J, Shambaugh J. The Geography of Prosperity. In: Nunn R, Shambaugh J, eds. Place-Based Policies for Shared Economic Growth. Washington, D.C. : Brookings Institution; 2018.
16. Ramaswamy S, Manyika J, Pinkus G, et al. Making it in America: Revitalizing US Manufacturing: McKinsey & Company Global Institute,; 2017.
17. Currie J, Schwandt H. Mortality inequality: the good news from a county-level approach. Journal of Economic Perspectives 2016;30:29-52.
18. United States Census Bureau. Quarterly Workforce Indicators. (Accessed January 19, 2018 at https://lehd.ces.census.gov/doc/QWI_101.pdf)
19. Goldstein A. Janesville: An American Story. New York: Simon & Schuster; 2017.
20. Rubenstein JM. The Changing U.S. Auto Industry-A Geographical Analysis. London: Routledge; 1992.
21. Kline P. Impact of juvenile curfew laws on arrests of youth and adults. American Law and Economics Review 2011;14:44-67.

© 2019 Venkataramani AS et al. *JAMA Internal Medicine*.

22. Goodman-Bacon A. Difference-in-differences with variation in treatment timing. NBER Working Paper 25018 2018.
23. Jacobson LS, LaLonde RJ, Sullivan DG. Earnings losses of displaced workers. American Economic Review 1993;83:685-709.
24. Sullivan D, von Wachter T. Job displacement and mortality: an analysis using administrative data. Quarterly Journal of Economics 2009;124:1265-306.
25. Borusyak K, Jaravel X. Revisiting event study designs, with an application to the estimation of the marginal propensity to consume. Mimeo, Harvard University 2017.
26. Angrist JD, Pischke J-S. Mastering Metrics: The Path from Cause to Effect. Princeton: Princeton University Press; 2015.
27. Bertrand M, Dulfo E, Mullainathan S. How much should we trust differences-in-differences estimates? Quarterly Journal of Economics 2004;119:249-75.
28. Greene W. The behavior of the maximum likelihood estimator of limited dependent variable models in the presence of fixed effects. Economic Journal 2004;7:98-119.
29. Sommers BD. State Medicaid expansions and mortality, revisited: a cost-benefit analysis. American Journal of Health Economics 2017;3:392-421.
30. Cameron AC, Miller DM. A practitioner's guide to cluster-robust inference. Journal of Human Resources 2015;50:317-72.
31. Cameron AC, Gelbach JB, Miller DL. Bootstrap-based improvements for inference with clustered errors. The Review of Economics of Statistics 2008;90:414-27.
32. Heaney CA, Israel BA, House JS. Chronic job insecurity among automobile workers: effects on job satisfaction and health. Social Science and Medicine 1994;38:1431-7.
33. Ganong P, Shoag DW. Why has regional income convergence in the U.S. declined? Journal of Urban Economics 2017;102:76-90.
34. Winkler R, Johnson KM, Cheng C, Beaudoin J, Voss PR, Curtis KJ. Age-Specific Net Migration Estimates for US Counties, 1950-2010. University of Wisconsin - Madison. (Accessed February 14, 2019 at https://netmigration.wisc.edu/.)
35. Greenland A, Lopresti J, McHenry P. Import competition and internal migration. The Review of Economics of Statistics 2019;101:44-59.
36. Wooldridge JM. Econometric Analysis of Cross Section and Panel Data. Cambridge, MA: MIT Press; 2010.

© 2019 Venkataramani AS et al. *JAMA Internal Medicine*.

eTable 1. Automotive Assembly Plants in Operation as of 1999, Location, and Closure Dates

	Company	Location	Date closed
**Plants included in main sample**			
1	AM General	Mishawaka, IN	
2	Autoalliance	Flat Rock, MI	
3	BMW	South Greer, SC	
4	DaimlerChrysler	Belvidere, IL	
5	DaimlerChrysler	Detroit, MI	July 2017
6	DaimlerChrysler	Gaffney, SC	
7	DaimlerChrysler	Detroit, MI	
8	DaimlerChrysler	Sterling Heights, MI	
9	DaimlerChrysler	Fenton, MO	July 2009
10	DaimlerChrysler	Fenton, MO	October 2008
11	DaimlerChrysler	Toledo, OH	
12	DaimlerChrysler	Toledo, OH	
13	DaimlerChrysler	Vance, AL	
14	DaimlerChrysler	Warren, MI	
15	Ford	Dearborn, MI	February 2004
16	Ford	Hapeville, GA	October 2006
17	Ford	Chicago, IL	
18	Ford	Dearborn, MI	
19	Ford	Detroit, MI	
20	Ford	Louisville, KY	
21	Ford	Lorain, OH	December 2005
22	Ford	Louisville, KY	
23	Ford	Wayne, MI	
24	Ford	Avon Lake, OH	
25	Ford	Norfolk, VA	June 2007
26	Ford	Hazelwood, MO	March 2006
27	Ford	Wayne, MI	
28	Ford	Wixom, MI	May 2007
29	General Motors	Lansing, MI	May 2005
30	General Motors	Bowling Green, KY	
31	General Motors	Detroit, MI	
32	General Motors	Doraville, GA	September 2008
33	General Motors	Flint, MI	
34	General Motors	Roanoke, IN	
35	General Motors	Janesville, WI	April 2009
36	General Motors	Lansing, MI	March 2006
37	General Motors	Lansing, MI	
38	General Motors	Lansing, MI	
39	General Motors	Warren , OH	
40	General Motors	Moraine, OH	December 2008
41	General Motors	Lake Orion, MI	
42	General Motors	Pontiac, MI	September 2009
43	General Motors	Spring Hill, TN	
44	General Motors	Wentzville, MO	
45	Honda	East Liberty, OH	
46	Honda	Marysville, OH	
47	Nissan	Smyrna, TN	
48	Subaru	Lafayette, IN	
49	Toyota	Georgetown, KY	
50	Toyota	Princeton, IN	
51	Freightliner/Sterling	Cleveland, NC	

© 2019 Venkataramani AS et al. *JAMA Internal Medicine*.

52	Freightliner	Mt. Holly, NC	
53	International	Springfield, OH	
54	Kenworth	Chillicothe, OH	
55	Mack/Volvo	Dublin, VA	
56	Mack	Winnsboro, SC	November 2002
57	Mack	Macungie, PA	
58	Peterbilt	Madison, TN	December 2009
**Plants not included in main sample**			
59	DaimlerChrysler	Newark, DE	December 2008
60	Ford	Edison, NJ	February 2004
61	Ford	Claycomo, MO	
62	Ford	St. Paul, MN	December 2011
63	General Motors	Linden, NJ	April 2005
64	General Motors	Baltimore, MD	May 2005
65	General Motors	Arlington, TX	
66	General Motors	Kansas City, KS	
67	General Motors	Oklahoma City, OK	February 2006
68	General Motors	Shreveport, LA	August 2012
69	General Motors	Wilmington, DE	July 2009
70	Mitsubishi	Normal, IL	November 2015
71	Nummi	Fremont, CA	
72	Freightliner/ Western Star	Portland, OR	
73	International	Garland, TX	May 2013
74	Kenworth	Renton, WA	
75	Peterbilt	Denton, TX	

Notes: List includes all plants involved in manufacturing of automobile, truck, SUV, and medium-heavy trucks identified using the search strategy described in Section 1. Plants included in the main sample are those situated within the 30 commuting zones with at least one manufacturing county (see Section 2 of this Supplemental Appendix for details on how manufacturing counties were defined). Of note, we identified four plants that opened after 2000 in commuting zones that previously did not have an automotive assembly plant: Honda (2001) in Lincoln, AL; Hyundai (2005) in Montgomery, AL; Nissan (2003) in Canton, MS; and Toyota (2006) in San Antonio, TX. These plants were not included in our analyses.

© 2019 Venkataramani AS et al. *JAMA Internal Medicine*.

eTable 2. Event Study Estimates from Figure 2 of Main Text, Including Alternate Calculation of Standard Errors

**Independent Variable**	**Estimates**
**Closure, -5 years and prior**	**0.479**
	[-2.377, 3.335]
	p=0.734
	wild p = 0.765
**Closure, -4 years**	**0.941**
	[-1.164, 3.047]
	p=0.368
	wild p = 0.470
**Closure, -3 years**	**-0.508**
	[-2.163, 1.148]
	p=0.537
	wild p = 0.422
**Closure, -2 years**	**0.504**
	[-1.872, 2.880]
	p=0.668
	wild p = 0.685
**Closure, -1 years**	**[REF]**
**Closure, +0 years**	**0.311**
	[-2.488, 3.109]
	p=0.8222
	wild p = 0.852
**Closure, +1 years**	**1.572**
	[-2.946, 6.090]
	p=0.482
	wild p = 0.518
**Closure, +2 year**	**3.555**
	[-0.805, 7.915]
	p=0.106
	wild p = 0.159
**Closure, +3 years**	**5.023**
	[0.819, 9.226]
	p=0.021
	wild p <0.001

© 2019 Venkataramani AS et al. *JAMA Internal Medicine*.

**Closure, +4 years**	**7.409**
	[3.121, 11.70]
	p=0.001
	wild p <0.001
**Closure, +5 years**	**8.635**
	[2.615, 14.66]
	p=0.006
	wild p = 0.016
**Closure, +6 years**	**7.668**
	[2.911, 12.42]
	p=0.003
	wild p = 0.008
**Closure, +7 years and onward**	**8.201**
	[2.097, 14.30]
	p=0.010
	wild p = 0.032
N (county-years)	2,016
N (counties)	112

Notes: Coefficient estimates from the main event study model for the primary outcome of opioid overdose mortality rates (per 100,000 individuals aged 18-65 years). All models include county and calendar year fixed effects (see main text and Section 4 of this Supplemental Appendix for further details on the event study specification). Point estimates for each event time binary variable are bolded. 95% CI, adjusting for clustering at the commuting zone level, are in square brackets. Below these are *p*-values based on cluster-corrected Huber-White standard errors. Wild cluster bootstrap *p-values* are denoted by "wild p." Section 5B of this Supplement provides further details on statistical inference using the wild-cluster bootstrap-t method.

© 2019 Venkataramani AS et al. *JAMA Internal Medicine*.

eFigure 1. US Counties by Quintile of the Manufacturing Share of Workforce in 2000

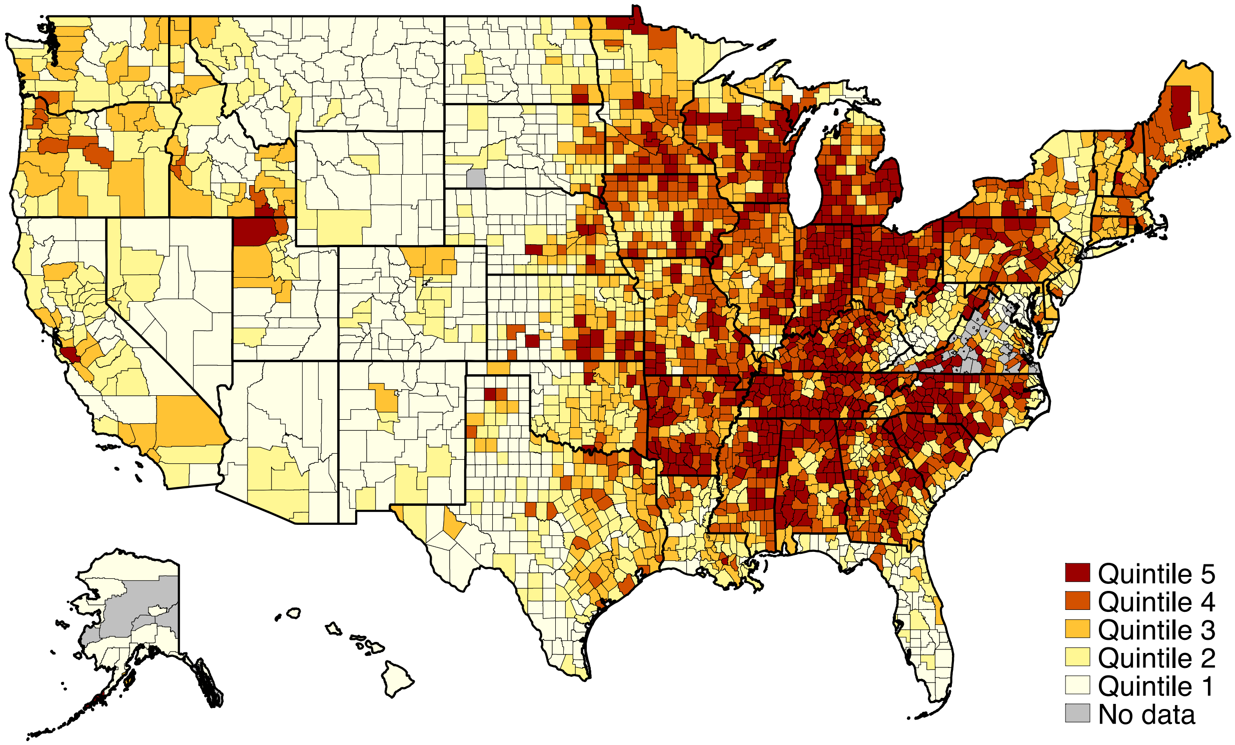

Notes: U.S. counties by quintiles of the share of employed residents above the age of 16 working in the manufacturing sector, based on data from the 2000 Decennial Census. Our main study sample consisted of all top-quintile counties located in commuting zones with at least one automotive assembly plant in operation as of 1999 (N=112 counties, see Figure 1 of main text).

© 2019 Venkataramani AS et al. *JAMA Internal Medicine*.

eFigure 2. Trends in Share of Sample Manufacturing Counties Exposed to Commuting Zone Automotive Assembly Plant Closure

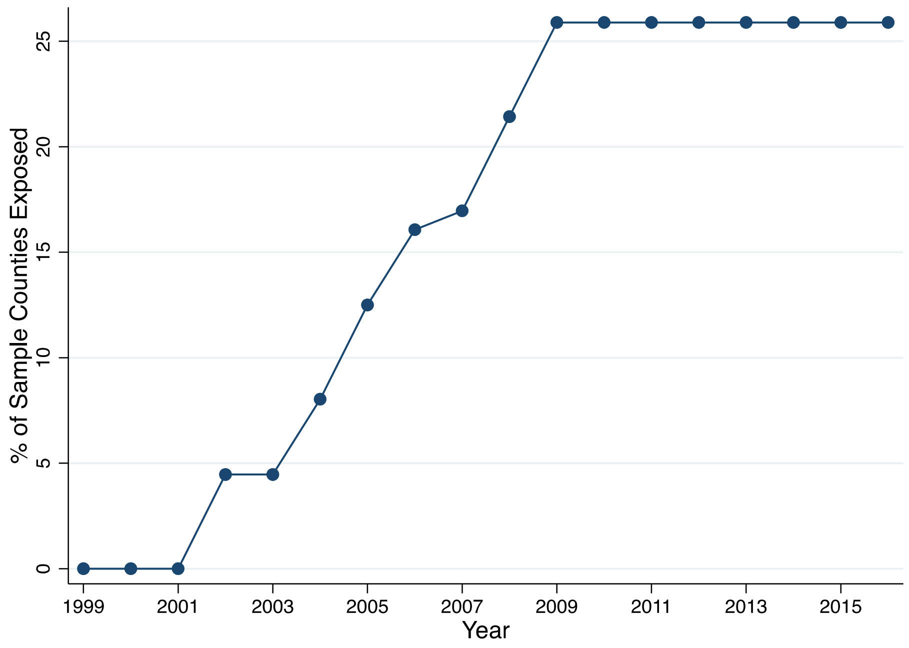

Notes: Trend in the percentage of the 112 sample counties exposed to plant closures over time. The y-axis denotes the cumulative percentage of manufacturing counties in the study sample that were exposed to an automobile assembly plant closure. At the beginning of the study period in 1999, there were no counties exposed to plant closure. By the end of the study period in 2015, 29 counties, located in 10 commuting zones, had been exposed.

© 2019 Venkataramani AS et al. *JAMA Internal Medicine*.

eFigure 3. Adjusted Difference-in-Differences Estimates for Overall Drug Overdose Mortality

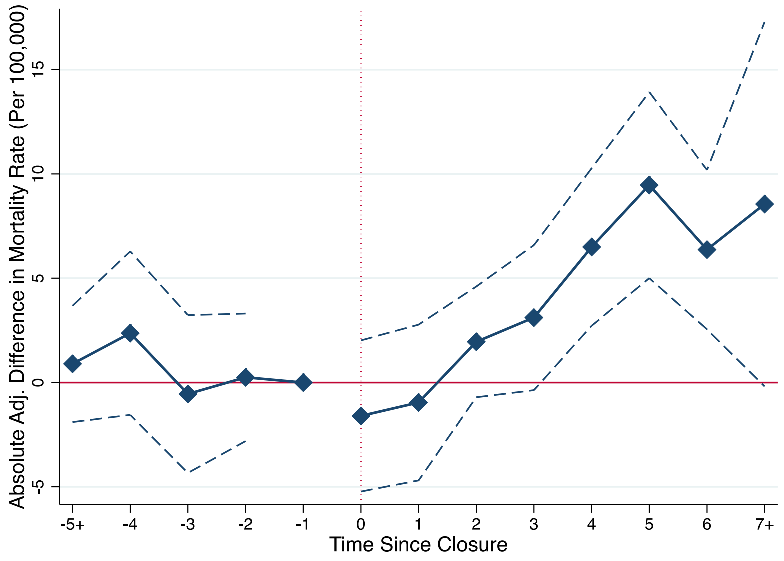

Notes: Coefficient estimates and 95% CIs from models identical to those estimated in **Figure 2** of the main text except here the main outcome is overall drug overdose mortality.

© 2019 Venkataramani AS et al. *JAMA Internal Medicine*.

eFigure 4. Adjusted Difference-in-Differences Estimates for Nonwhites, Stratified by Sex-Age Subgroups

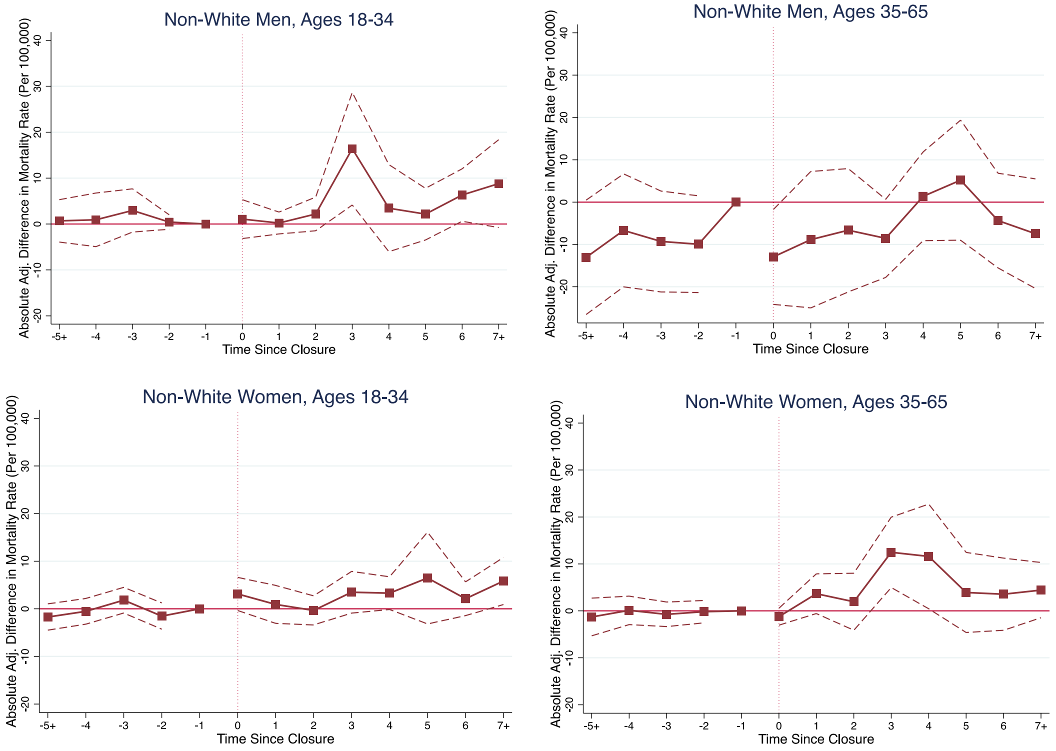

Notes: Coefficient estimates and 95% Cis from models identical to those estimated in **Figure 4** of the main text except here populations of interest are non-white subgroups. See **Figure 2** and **Figure 4** notes for further details.

© 2019 Venkataramani AS et al. *JAMA Internal Medicine*.

eFigure 5. Adjusted Difference-in-Differences Estimates by Class of Opioid Stratified by Demographic Subgroup

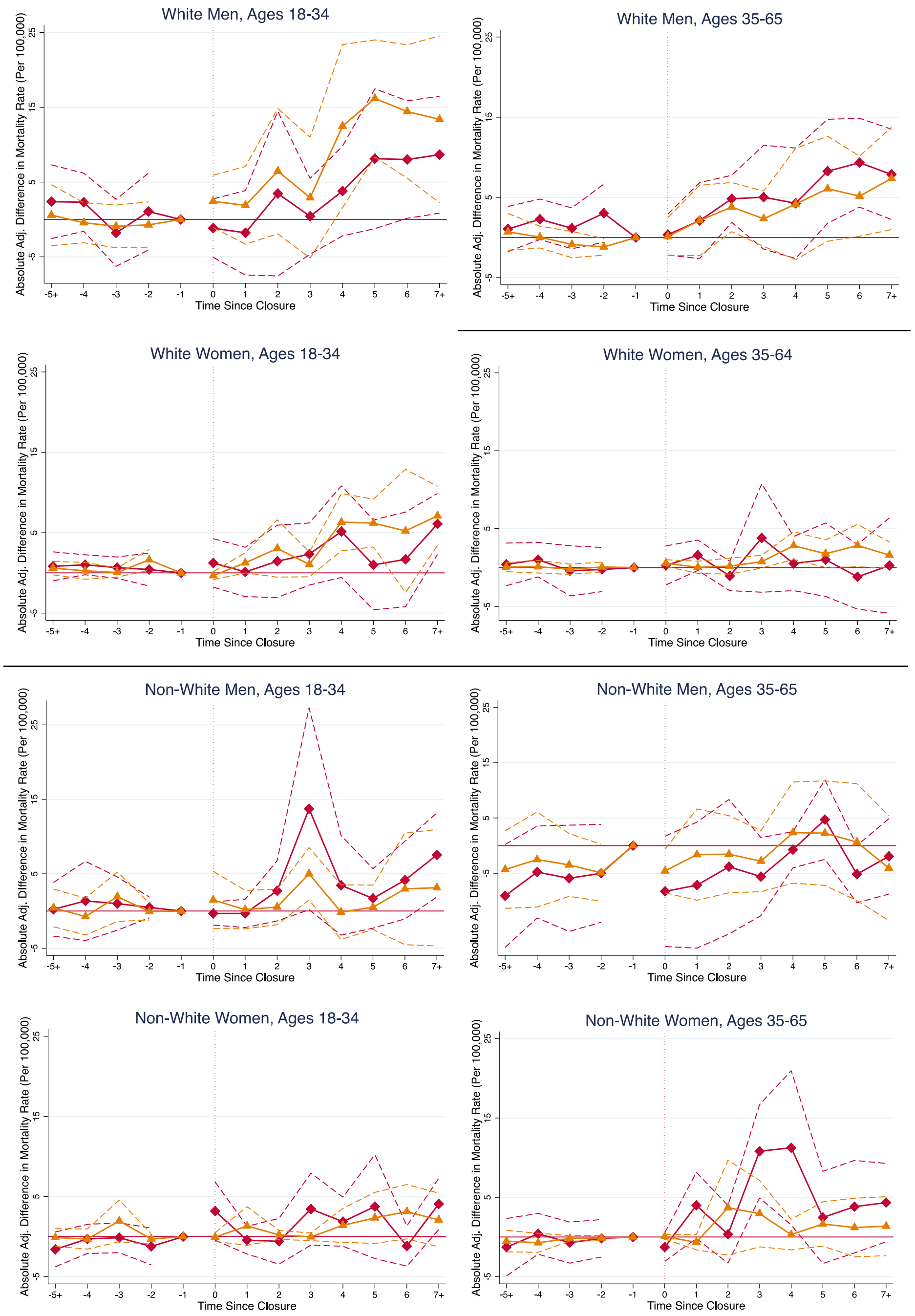

Notes: Coefficient estimates and 95% Cis from models identical to those presented in **Figure 3** of the main text, except here the samples are stratified by race/ethnicity-age-sex subgroups. See **Figure 2** and **3** notes for further details.

© 2019 Venkataramani AS et al. *JAMA Internal Medicine*.

eFigure 6. Adjusted Difference-in-Differences Estimates, Modelling Overdose Deaths as a Count Variable

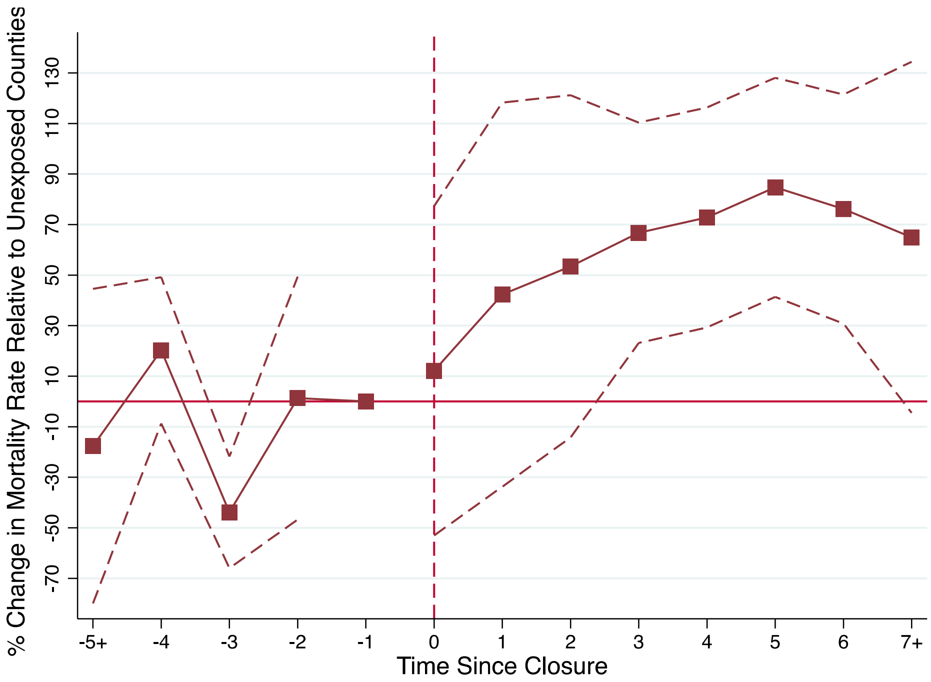

Notes: Coefficient estimates and 95% CIs from a generalized linear model where the number of opioid overdose deaths was specified as the dependent variable. We assumed a negative binomial distribution and a log-link, and the county-year population was specified as the exposure. The coefficient estimates can be interpreted as relative changes in mortality rates (see Section 5A of this Supplement for further details). Covariates and sample size are identical to the main specification (see main text and **Figure 2** notes for further details).

© 2019 Venkataramani AS et al. *JAMA Internal Medicine*.

eFigure 7. Adjusted Difference-in-Differences Estimates for an Expanded Sample That Includes Manufacturing Counties From Each Commuting Zone Excluded From Main Sample

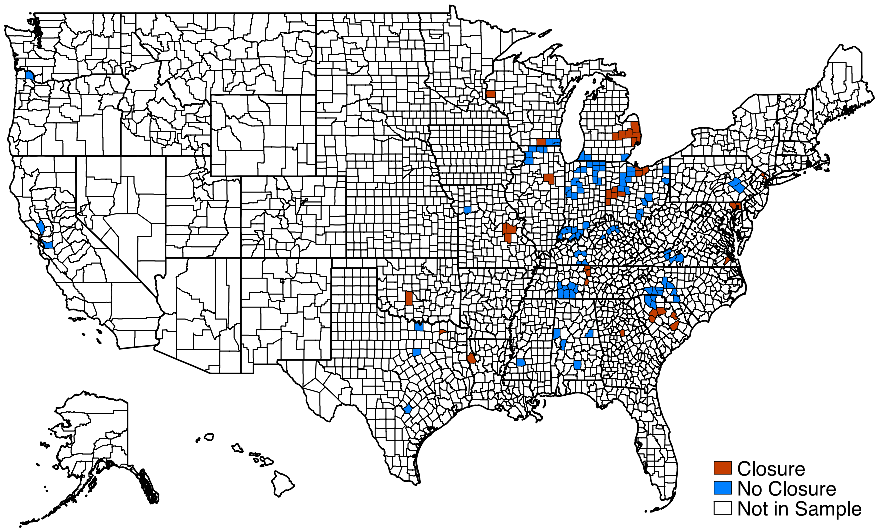

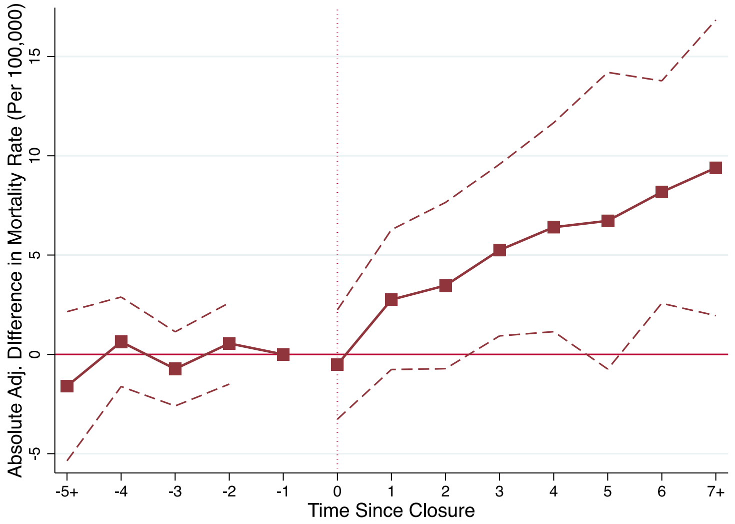

Notes: Coefficient estimates and 95% CIs from the same statistical model as in **Figure 2** of the main text, except here we used an expanded sample of counties, comprising all counties from the main sample as well as the highest manufacturing share county from each commuting zone excluded from the main sample. See Section 5C for further details. The sample included 2,358 county-year observations (37 exposed and 94 unexposed counties across 48 commuting zones; top panel).

© 2019 Venkataramani AS et al. *JAMA Internal Medicine*.

eFigure 8. Adjusted Difference-in-Differences Estimates Using an Alternate Control Group

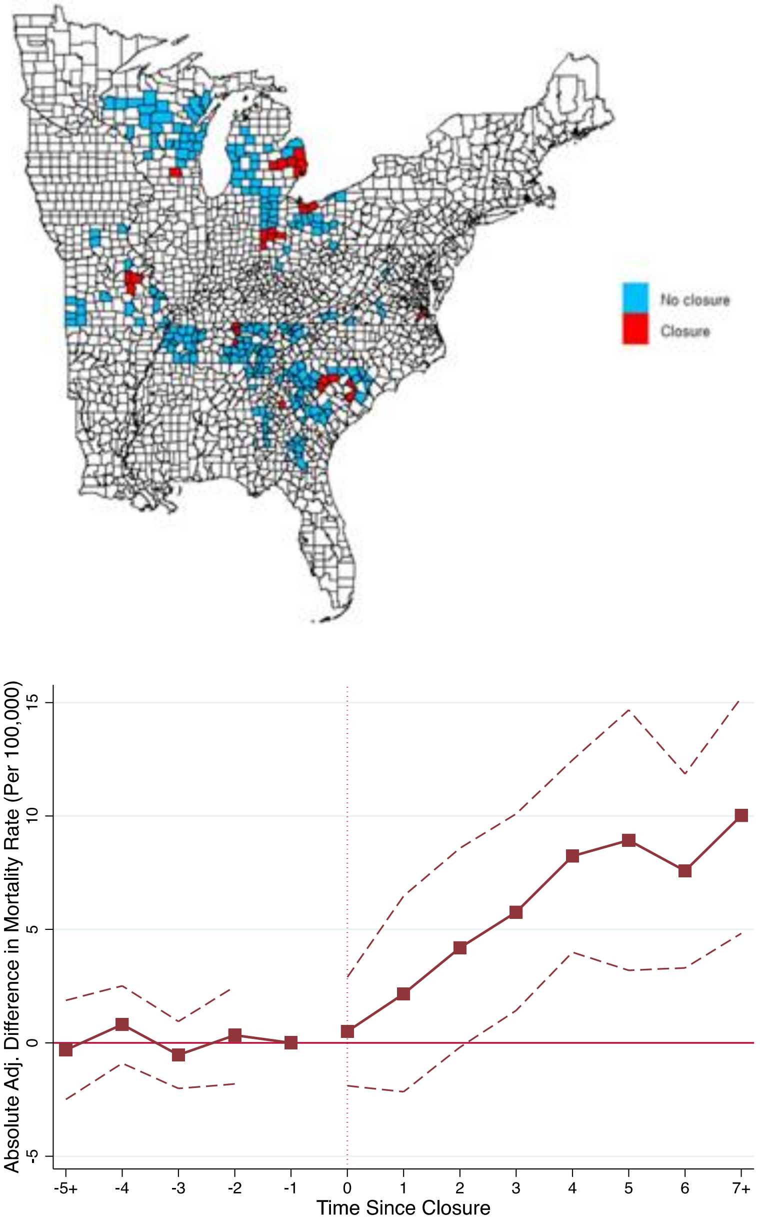

Notes: Coefficient estimates and 95% CIs from the same statistical model as in **Figure 2** of the main text, except here we used an alternate control group, which was comprised of manufacturing counties located in non-automotive commuting zones that were situated in the same states as commuting zones with automotive plant closures (top panel). Section 5D provides further details. The sample included 4,176 county-year observations (29 exposed and 203 unexposed counties across 97 commuting zones; top panel). Model included county and calendar year fixed effects.

© 2019 Venkataramani AS et al. *JAMA Internal Medicine*.

eFigure 9. Adjusted Difference-in-Differences Estimates in Nonmanufacturing Counties

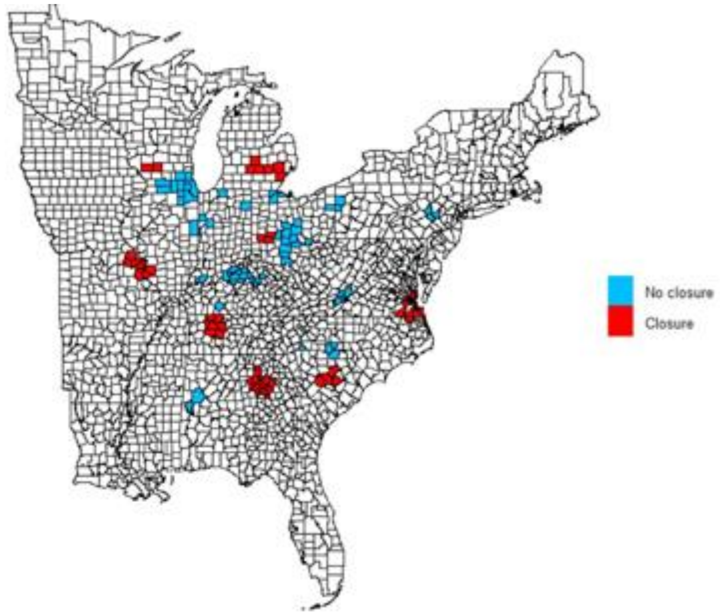

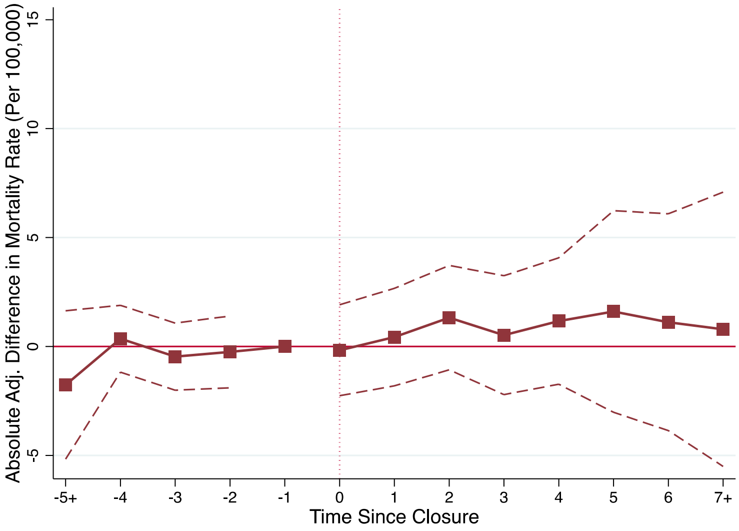

Notes: Coefficient estimates and 95% CIs from the same statistical model as in **Figure 2** of the main text, except here the sample consists of non-manufacturing counties, defined as counties in the bottom four quintiles nationally with respect to the share of workers in the manufacturing industry in 2000 (see Section 2 and Section 5E for further details). The sample included 2,052 county-year observations (52 exposed and 62 unexposed counties; top panel), all within the same 30 commuting zones as the main sample. Model included county and calendar year fixed effects. Of note, in fully interacted models, post-event coefficient estimations were jointly statistically significantly different for manufacturing vs. non-manufacturing counties ($F(8, 29) = 2.38$, p=0.04). Differences in the pre-event coefficient estimates were not statistically significantly different between these groups ($F(4, 29) = 1.11$, p=0.37).

© 2019 Venkataramani AS et al. *JAMA Internal Medicine*.

eFigure 10. Differences in Net Migration Rates by Exposure to Automotive Assembly Plant Closures

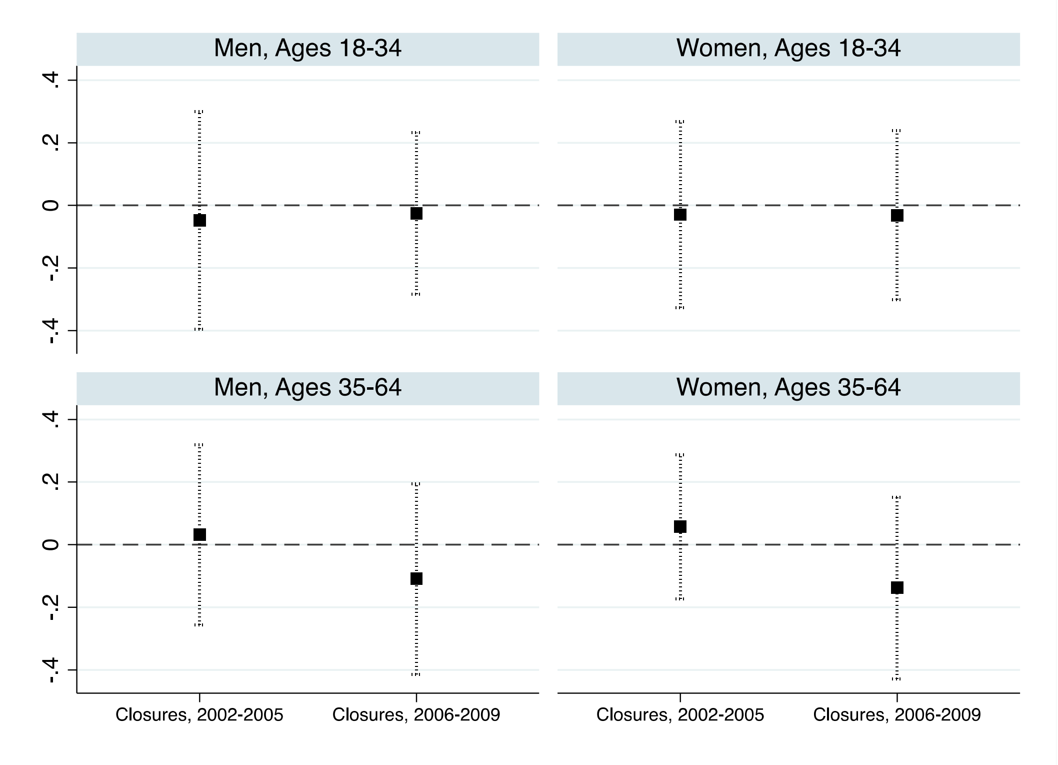

Notes: Each graph presents estimates from Equation 2 (see Section 5F of this Supplemental Appendix) for the demographic subgroup listed in the header. The sample size for each regression is 112 counties (the 29 exposed and 83 unexposed counties that form the main sample). "Closures, 2002-2005" and "Closures, 2006-2009" are binary indicators = 1 if that particular county experienced an automotive assembly plant closure in the given time period. Estimates represent the standard deviation change in county net migration rates. 95% CI are corrected for clustering at the CZ level. All estimates range between -0.13 to 0.05 s.d. of the dependent variable.

© 2019 Venkataramani AS et al. *JAMA Internal Medicine*.
